# BS-Seeker2: a versatile aligning pipeline for bisulfite sequencing data

**DOI:** 10.1186/1471-2164-14-774

**Published:** 2013-11-10

**Authors:** Weilong Guo, Petko Fiziev, Weihong Yan, Shawn Cokus, Xueguang Sun, Michael Q Zhang, Pao-Yang Chen, Matteo Pellegrini

**Affiliations:** 1 Center for Synthetic & Systems Biology, TNLIST, Tsinghua University, Beijing 100084, China; 2 Department of Molecular, Cell and Developmental Biology, University of California, Los Angeles, CA 90095, USA; 3 Department of Biological Chemistry, University of California, Los Angeles, CA 90095, USA; 4 Department of Chemistry and Biochemistry, University of California, Los Angeles, CA 90095, USA; 5 Zymo Research Corp, 17062 Murphy Ave, Irvine, CA 92614, USA; 6 Department of Molecular and Cell Biology, Center for Systems Biology, The University of Texas at Dallas, Richardson, TX 75080, USA; 7 Institute of Plant and Microbial Biology, Academia Sinica, Taipei 11529, Taiwan; 8 Institute for Genomics and Proteomics, University of California, Los Angeles, CA 90095, USA

**Keywords:** DNA methylation, Bisulfite sequencing aligner, WGBS, RRBS, BS Seeker, Bisulfite conversion failure, Galaxy toolshed

## Abstract

**Background:**

DNA methylation is an important epigenetic modification involved in many biological processes. Bisulfite treatment coupled with high-throughput sequencing provides an effective approach for studying genome-wide DNA methylation at base resolution. Libraries such as whole genome bisulfite sequencing (WGBS) and reduced represented bisulfite sequencing (RRBS) are widely used for generating DNA methylomes, demanding efficient and versatile tools for aligning bisulfite sequencing data.

**Results:**

We have developed BS-Seeker2, an updated version of BS Seeker, as a full pipeline for mapping bisulfite sequencing data and generating DNA methylomes. BS-Seeker2 improves mappability over existing aligners by using local alignment. It can also map reads from RRBS library by building special indexes with improved efficiency and accuracy. Moreover, BS-Seeker2 provides additional function for filtering out reads with incomplete bisulfite conversion, which is useful in minimizing the overestimation of DNA methylation levels. We also defined CGmap and ATCGmap file formats for full representations of DNA methylomes, as part of the outputs of BS-Seeker2 pipeline together with BAM and WIG files.

**Conclusions:**

Our evaluations on the performance show that BS-Seeker2 works efficiently and accurately for both WGBS data and RRBS data. BS-Seeker2 is freely available at http://pellegrini.mcdb.ucla.edu/BS_Seeker2/ and the Galaxy server.

## Background

DNA methylation is an important epigenetic mark that is involved in gene regulation, X-chromosome inactivation, imprinting and development. Next-generation sequencing of bisulfite converted DNA makes it possible to detect genome wide DNA methylation at base-pair resolution [1]. WGBS generates high quality DNA methylomes covering more than 90% of cytosines in the human genome, at a single base-pair resolution [2]. An alternative to WGBS is RRBS [3], which is becoming popular for studies with multiple samples. In RRBS, genomic DNA is first fragmented by enzymatic digestion (e.g. MspI) and followed by a size selection step to enrich the fragments for CpG islands. Additionally, double restriction-enzyme digestion methods may improve the coverage and accuracy of RRBS [4].

To date, several bisulfite-sequencing (BS) aligners have been developed. BS Seeker [5] was the first BS aligner based on a three-letter approach using a general purpose short read mapper, Bowtie [6]. Subsequently, similar tools were developed including Bismark [7], BRAT-BW [8] and MethylCoder [9]. BS aligners employing three-letter approaches perform *in silico* C-to-T conversion for both reads and reference sequences prior to mapping. Other BS aligners, such as BSMAP [10], RMAPBS [11] and GSNAP [12], employ wild-card approaches.

Most of these alignment tools are designed for WGBS, and only RRBSMAP [13] is tailored for RRBS by mapping adapter-trimmed reads around the restriction enzyme cutting sites. Tools such as Bismark can also map RRBS reads against the reference genome with the assistance of external tool for trimming adapters. However, these tools also attempt to map the reads to whole genome including regions where the reads would not be oriented from, leading to inefficient use of computational resources and increased mapping errors. Moreover, most of these aligners do not allow gapped alignment (e.g., RMAPBS, BRAT-BW). Bismark performs gapped mapping when using Bowtie2, but it only enables the ‘end-to-end’ mode. BSMAP can handle one continuous gap with up to three nucleotides.

Here we present BS-Seeker2, an updated version of BS Seeker. BS-Seeker2 is a bisulfite sequencing alignment tool that performs genome indexing, read alignment and DNA methylation levels calling for each cytosine. It supports both local and gapped alignment by integrating Bowtie2 [14], Bowtie [6], SOAP [15] and RMAP [16]. Various types of libraries are supported, including WGBS/RRBS, directional/non-directional library, single-end/paired-end sequencing, and user-defined enzyme cutting sites for variant versions of RRBS. BS-Seeker2 maps RRBS data efficiently and accurately by only indexing the reduced representation genome regions. BS-Seeker2 works with raw sequences and generates outputs for read alignments and methylation levels at single-base resolution. BS-Seeker2 also provides an option to remove reads with incomplete bisulfite conversion, reducing the overestimation of DNA methylation levels. Lastly, BS-Seeker2 is available through Galaxy [17] via the Toolshed (http://toolshed.g2.bx.psu.edu).

We compared the performance of BS-Seeker2 with Bismark and BSMAP on both real data and simulated data on mappability, mapping accuracy and computational CPU and RAM costs. Our results show that BS-Seeker2 is able to accurately and efficiently map reads from both WGBS and RRBS protocols. On real data, BS-Seeker2 in the local alignment mode maps more reads than the other aligners. By mapping to the reduced representation genome, BS-Seeker2 is more efficient and accurate than mapping to the whole genome.

## Implementation

### BS-Seeker2 as a pipeline for aligning bisulfite sequencing data

**Figure 1:**
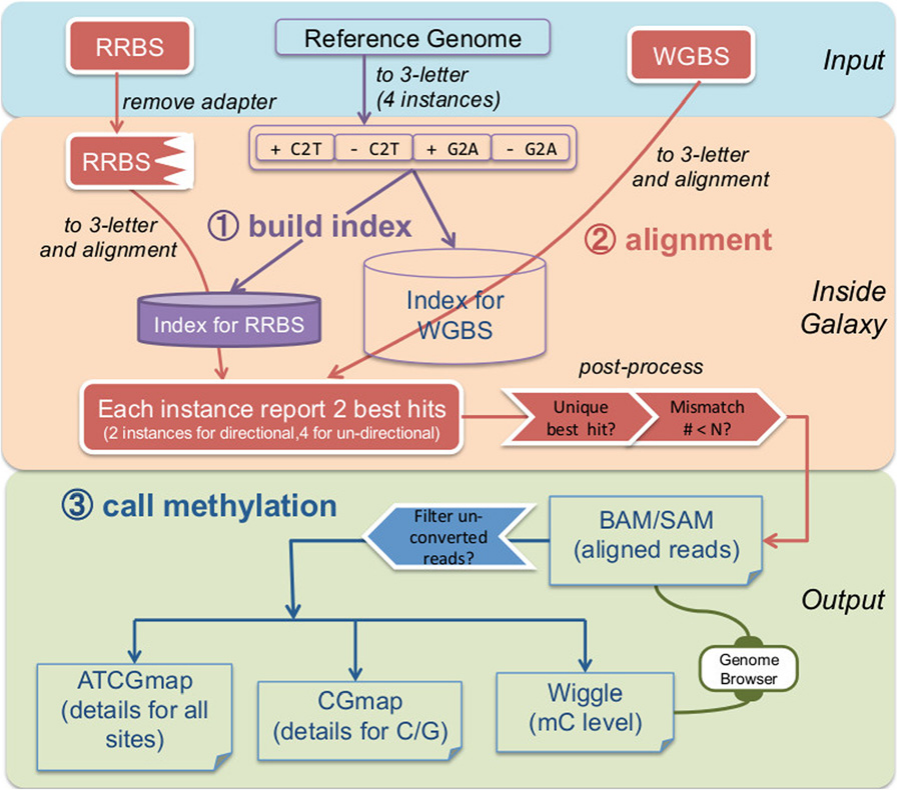
**The three main steps in the workflow of BS-Seeker2. (1)** Index-building. Indexes for RRBS and WGBS are built separately from a three-letter converted genome. Four index instances are built to account for the asymmetric bisulfite-conversion of the two strands and properties of non-directional libraries. **(2)** Aligning reads to the indexes. Both WGBS and RRBS reads are converted to three-letters prior to mapping. For RRBS, adapters should be removed first. Converted reads are mapped onto four index instances for non-directional libraries (two instances for directional libraries), and mapping to each index instance will report two best hits. Multiple hits and mismatch numbers are checked before being reported as alignment results. The C-to-T match is regarded as a mismatch in this step, and is checked by the mismatch criteria. **(3)** Calling methylation level for each site. The user can decide whether to filter the un-converted reads in this step. BS-Seeker2 provides detailed outputs (BAM/SAM, wiggle, CGmap and ATCGmap files). Both the wiggle file and the BAM file can be directly imported in a genome browser, such as IGV. BS-Seeker2 is also integrated into the Galaxy web interface platform.

BS-Seeker2 is implemented in Python, integrating steps of building indexes from reference genomes, mapping reads from various formats (qseq, fastq, fasta and pure sequence), and generating alignment results (BAM, SAM or BS-Seeker format) and methylation calls (wiggle format), which can be directly loaded into a genome browser, such as IGV [18] (Figure 1). Detailed mapping summaries for each cytosine (CGmap) and all covered positions (ATCGmap) are also reported for downstream analysis (Additional file 1: Supplementary Methods). BS-Seeker2 can be coupled with a variety of short read aligners with a three-letter approach. BS-Seeker2 is also highly customizable, as the user can choose alignment modes, and control almost all the parameters of utilized aligners.

### Gapped mapping and local alignment

**Figure 2:**
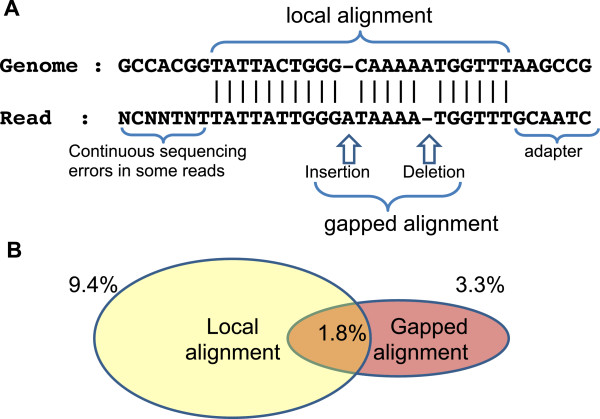
**Gapped alignment and local alignment. (A)** An example shows how gapped alignment and local alignment work and occurrence condition. **(B)** Venn chart shows the percentages of the total reads from real WGBS testing data set that could be mapped by gapped alignment or local alignment utilizing Bowtie2-local but not by Bowtie.

BS-Seeker2 takes advantage of Bowtie2’s gapped-mapping, and supports both ‘local’ and ‘end-to-end’ alignment modes. By using local alignment, BS-Seeker2 can effectively map reads with 3’ contamination of adapters (Figure 2A). In Illumina sequencing, reads sometimes contain continuous sequencing errors or missing base calls, probably caused by bubble in flow cell (22). BS-Seeker2 circumvents these problems by using local alignments to remove the mismatched nucleotides from the end of the reads to maximize the mappability. In order to quantify mappability improvements attributable to gapped-mapping and local alignments, we compared mapping results of BS-Seeker2 on real sequencing data by utilizing Bowtie and Bowtie2-local. The results showed that in the real testing data set for WGBS, compared to Bowtie, an extra 11% of total reads could be mapped by using the local alignment model of Bowtie2. Specifically, 3.3% of the total reads could be mapped by allowing indels (the gapped alignment) and 9.4% could be salvaged using the local alignment feature (Figure 2B).

### Masked genome for RRBS mapping

**Figure 3:**
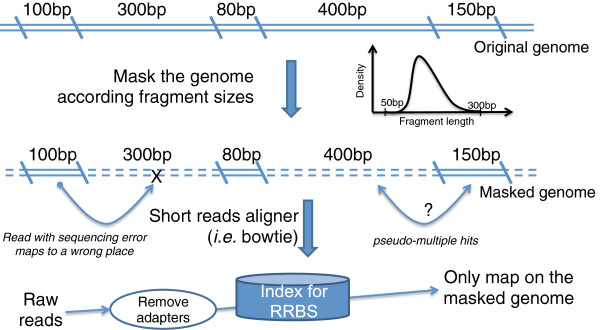
**A diagram illustrating how specific indexes are built for RRBS.** The original genome is cut by restriction enzyme(s) into fragments. Fragments with lengths in a specific range (e.g. from 50 bp to 300 bp) are selected, whereas unselected regions are masked. The unmasked genome is used for building the index.

BS-Seeker2 builds special index for RRBS libraries, which results in improvements of mapping speed, mappability and mapping accuracy and less memory usage (Figure 3). RRBS libraries are generated by restriction enzyme (i.e. MspI) digestion and the subsequent selection of fragment sizes typically ranging from 40 bp to 220 bp, corresponding to less than 5% of the entire genome. To model this we mask the reference genome in silico based on restriction sites (e.g., C’CGG for MspI). Genomic regions not falling within the size-selected RRBS fragments are masked and only unmasked regions are indexed (RR genome). There are four main advantages for mapping against the RR genome instead of the whole genome. First, it reduces the size of the pre-built index. Taking the reference genome mouse mm9 as an example, the *.ebwt files built by Bowite are over ~12G bytes for the whole genome, but only ~0.3G bytes for the masked genome. Second, it accelerates the alignment step. Mapping to RR genome is about 3 times faster than mapping to the whole genome (Table 1), as the masked genome represents a much smaller search space. Third, it increases the mapping accuracy. Masked genomes help reduce spurious mapping when the reads contain sequencing errors (Figure 3). Based on our simulated error-containing data, accuracy for mapping to the RR genome (99.33%) is higher compared to mapping to the whole genome (97.92%) (Table 1). Lastly, it keeps reads that would have pseudo-multiple hits when mapping to whole genome. A pseudo-multiple hit occurs when a read coming from the RR genome has another best match in the masked regions. In the simulated error-free data, pseudo-multiple hits are avoided when the reads are mapped to the RR genome, resulting in higher mappability (74.04%) than mapped to the whole genome (72.52%) (Table 1).

**Table 1:** Performance comparisons for mapping simulated RRBS reads to RR and WG indexes

	**Error-free**			**Error-containing**		
	**To RR**		**To WG**	**To RR**		**To WG**
Mappability	74.04%	←	72.52%	74.41%	←	72.95%
User time	1m23s	←	4m18s	1m20s	←	4m37s
Accuracy	100.00%		100.00%	99.33%	←	97.92%

100 k reads of length 50 bp are simulated from the RR genome. Mapping is done using BS-Seeker2 (Bowtie). Mapping to the reduced represented (RR) index is much faster than mapping to the whole genome (WG) index. For error-free samples, the mappability to RR is higher than WG by avoiding pseudo-multiple hits. For error-containing samples, mapping to the RR index will result in higher accuracy than mapping to the WG index. Arrows indicate the improvement directions.

### Filtering reads with incomplete bisulfite conversion

**Figure 4:**
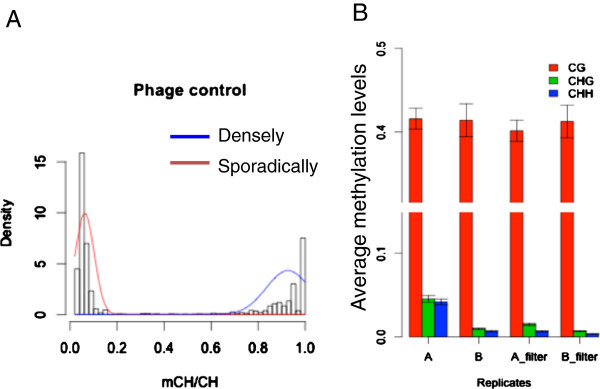
**Filtering reads with incomplete bisulfite conversion. (A)** Distribution of the unconverted ratio of CH sites (H = A, C, T) in phage DNA reads which has at least one CH site unconverted. Phage DNA is free of DNA methylation and used as a control. The distribution chart indicates two different categories: sporadic (red) and dense (blue) methylation. BS-Seeker2 provides an option for removing reads with dense non-CpG methylation. **(B)** Filtering un-converted reads makes the methylation levels of two technical replicates more similar. Error bar, SD.

High bisulfite conversion rate is a critical factor for accurately estimating the methylation levels. Incomplete bisulfite conversion may lead to an overestimation of the methylation level. BS-Seeker2 provides a computational solution to remove reads with incomplete bisulfite conversion (Additional file 1: Supplementary Methods). Unmethylated phage DNA is often spiked into the samples as a control, to measure bisulfite conversion rates. We analysed the methylation pattern of the phage reads and observed two groups of reads from the distribution of unconverted cytosine sites: sporadically distributed and densely distributed (Figure 4A). The sporadic group could be due to random un-conversion failure, or from T-to-C sequencing errors. The dense group is a set of reads that are almost entirely un-converted, potentially caused by the formation of secondary structure. We found that 82% of un-converted non-CpG sites were in the dense group, and only 18% were in the sporadic group. The same pattern was also observed on the mouse DNA data (Additional file 1: Figure S1). BS-Seeker2 provides an optional function to remove reads with densely un-converted non-CpG sites. To validate the feasibility, we mapped RRBS reads of two technical replicates (from the same mouse sample but different libraries), denoted as data sets A and B. The calculated methylation levels of non-CpG contexts show about 5-fold difference between the two replicates (Figure 4C). After removing the potentially un-converted reads, the methylation level gaps of non-CpG contexts were narrowed (to about 2-fold). However, this option is not suggested for samples with highly methylated non-CpG context, as it might reject bona fide methylated reads.

### CGmap and ATCGmap files

We defined new file formats for the representation of DNA methylomes, CGmap and ATCGmap (Additional file 1: Supplementary Methods). The DNA methylation levels and read counts for both CpG and CpH sites are shown in CGmap file. ATCGmap file includes read counts for all mapped sites and both strands. Both CGmap and ATCGmap files provide detailed mapping results, and are convenient for downstream analysis.

### Integration with galaxy

We have integrated BS-Seeker2 into Galaxy to generate a user-friendly bisulfite sequencing read aligner. Users may can use our Galaxy server (http://galaxy.mcdb.ucla.edu) (Additional file 1: Figure S2), or install BS-Seeker2 in their local Galaxy server via Galaxy Toolshed (http://toolshed.g2.bx.psu.edu).

## Results

### Evaluations of different BS aligners

We evaluated BS-Seeker2 against two other popular BS aligners, Bismark and BSMAP. Both BS-Seeker2 and Bismark are implemented based on the three-letter approach, whereas BSMAP is based on the wild-card approach. We compared them on both WGBS and RRBS data. To estimate the mapping accuracy, we test the three tools on two simulated data sets that are error-free and error-containing, respectively. Three models of BS-Seeker2 were used: BS-Seeker2-local (local alignment mode of Bowtie2), BS-Seeker2-e2e (end-to-end alignment mode of Bowite2) and BS-Seeker2-Bowtie (utilizing Bowtie). Since Bismark supports Bowtie2 only with the ‘end-to-end’ alignment model, we tested it in two modes: Bowite2-e2e and Bowtie.

Parameters. For all the evaluations of BS aligners (BS-Seeker2, Bismark v0.7.7, BSMAP v2.73), 100 k reads (1x100 bp/ 2x60 bp) were used. Up to 5 mismatches were allowed for mapping, except for Bismark-Bowtie2-e2e (end-to-end model of Bowtie2), which does not provide such parameters. Both BS-Seeker2 and Bismark utilize Bowtie/Bowtie2 for mapping, and the main parameters passed to the short read aligners are identical. The exact commands used in testing are listed in Additional file 1: Supplementary Methods. The mapping runs were performed on a Linux server with 12 cores (Intel(R) Xeon(R) CPU, X5650, 2.67GHz) and with 48 Gb RAM running 64bit Red Hat 4.4.7-3.

RRBS related issues. In the comparisons of mapping RRBS reads, BS-Seeker2 mapped the reads against the index of the reduced represented (RR) genome. Both Bismark and BSMAP map reads against the whole genome regardless of the RRBS fragment lengths. BS-Seeker2 and BSMAP have built-in functions to remove adapters, while Bismark does not. As a result we performed an additional step for adapter trimming (by running trim_galore) for Bismark.

Evaluation criteria. We evaluated the performance of the BS aligners on four criteria: 1) mappability, the percentage of reads that are uniquely mapped against all reads; 2) mapping accuracy, the percentage of the correctly mapped reads against all the uniquely mapped reads (only for simulated data); 3) time, the CPU time used for alignment, which is calculated as the total CPU seconds cost by the whole process in user mode; 4) RAM usage, the maximum cost of random-access memory for a whole task.

### Bisulfite sequencing data–real and simulated data

The real data sets are single-end reads generated from mouse/human bisulfite libraries. The WGBS data is downloaded from published data sets, SRR299053 (single-end, mouse) (21) and SRR306438 (paired-end, human) [19]. The RRBS data is from our unpublished mouse RRBS library. In order to measure the mapping accuracy, we also generated simulated reads (Additional file 1: Supplementary Methods). All the simulated reads are randomly generated from the reference genome (mm9/hg18) assuming directional libraries with read lengths same with those of the real data sets. RRBS reads are generated from fragments with lengths ranging between 40 bp and 250 bp. For reads shorter than 100 bp, the adapter sequence “AGATCGGAAGAGCACACGTCTGAACTCCAGTCA” was added to the 3’ end. Two kinds of simulated sequences were generated: error-free and error-containing reads. The error-free simulated reads are faithfully converted and have no sequencing error. The simulated error-containing reads are converted with 1% failure, and sequencing errors by cycles are also added. The error rate per cycle follows the distribution of sequencing error rates in the real data (Additional file 1: Supplementary Methods). All the datasets used for testing aligners are available on the website of BS-Seeker2.

### Performance on WGBS mapping

Generally, for single-end data of WGBS library, the performance of BS-Seeker2 is comparable with both Bismark and BSMAP (Table 2). BS-Seeker2 has the similar mappability and mapping accuracy with Bismark and BSMAP on simulation data. In real data, BS-Seeker2 with local alignment mode has the highest mappability (83.80%), which is about 10% higher than any alignment mode used in Bismark (73.15% for Bowtie) and BSMAP (72.84%). This suggests that local alignments are more suitable for real data, which could have more sequencing errors (such as continuous errors, indels, adapters etc.) than simulated data. Indels, continuous errors and adapters were not considered in the simulated data sets. Results also showed that BS-Seeker2-Bowtie requires the less memory than Bismark and BSMAP (Additional file 1: Figure S3), and the speed is improved over Bismark (Additional file 1: Figure S4). Both BS-Seeker2 and Bismark integrating with Bowtie2 require more time and more memory than they were with Bowtie, as Bowtie2 would require more resources for multiple-seed strategy and searching indels.

**Table 2:** Performance comparison of BS aligners on WGBS data

	**BS-Seeker2**			**Bismark**		**BSMAP**
**WGBS**	**Bowtie2**		**Bowtie**	**Bowtie2**	**Bowtie**	
	**local**	**e2e**		**(e2e)**		
Simulation: error-free						
map	91.65%	91.50%	91.65%	87.78%	91.65%	91.81%
acc	100.00%	100.00%	100.00%	100.00%	100.00%	100.00%
Simulation: error-containing						
map	91.62%	90.51%	91.69%	86.90%	91.64%	91.90%
acc	99.22%	99.73%	99.82%	99.86%	99.80%	99.82%
Real data						
map	83.80%	72.94%	71.89%	70.31%	73.15%	72.84%

In this table, BS-Seeker2 maps the reads to whole genome. map = mappability, acc = accuracy, local = local alignment model of Bowtie2, e2e = end-to-end alignment model of Bowtie2.

Similarly, for the paired-end data of WGBS library, both BS-Seeker2 and Bismark map more reads than BSMAP on simulation data, also with relatively higher accuracy (Additional file 1: Table S1). Among the three aligners, BS-Seeker2 requires the least memory (Additional file 1: Figure S3). The local alignment mode of BS-Seeker2 has the highest mappability. Different from that of single-end data, our comparisons showed that none of the three aligners achieve 100% accuracy when mapping the error-free simulated paired-end reads. As the searching space when aligning the mate pairs is quite large, it is possible that only suboptimal hits are reported in order to improve efficiency.

### Performance on RRBS mapping

BS-Seeker2 outperform the other aligners on RRBS data (Table 3). For the error-free data, BS-Seeker2 has the highest mappability and 100% accuracy. Bismark mapped reads to the whole genome with lower mappability than BS-Seeker2, as a result of the pseudo-multiple hits issue. BSMAP is the only aligner whose mapping accuracy doesn’t reach 100% when mapping simulated error-free reads. For error-containing simulated data, BS-Seeker2-Bowtie shows improved mappability and accuracy compared to Bismark-Bowtie and higher accuracy than BSMAP. As the 3’ end of reads tends to have l more sequencing errors, it’s not easy to remove all the adapters, since this portion tends to be of lower quality in real data, making reads with 3’ un-trimmed adapters difficult to align. BS-Seeker2 using the local alignment mode of Bowtie2 provides an effective way to map these reads. An appropriately broad range for fragment lengths of RR genome is suggested for BS-Seeker2 to optimize the mappability (Additional file 1: Supplementary Method and Figure S5).

**Table 3:** Performance comparison of BS aligners on RRBS data

	**BS-Seeker2**			**Bismark**		**BSMAP**
**RRBS**	**Bowtie2**		**Bowtie**	**Bowtie2**	**Bowtie**	**(RRBSMAP)**
	**local**	**e2e**		**(e2e)**		
Simulation: error-free						
map	78.29%	78.02%	78.29%	72.51%	78.08%	78.63%
acc	100.00%	100.00%	100.00%	100.00%	100.00%	99.82%
Simulation: error-containing						
map	79.18%	78.42%	78.72%	71.36%	78.17%	79.10%
acc	98.11%	98.59%	99.02%	99.61%	98.82%	98.81%
Real data						
map	64.45%	48.78%	47.29%	44.24%	46.89%	45.64%

map = mappability, acc = accuracy, local = local alignment mode, e2e = end-to-end alignment mode. In this table, BS-Seeker2 maps the reads to RR genome (fragment lengths ranging 20 bp ~ 400 bp).

### Feature comparisons

BS-Seeker2 has been improved from BS-Seeker, by integrating variable aligners, supporting both WGBS and RRBS, and allowing mapping both single-end and paired-end reads, supporting all major input formats and output formats. For a clear view on the improvements of BS-Seeker2, a comparison table on the supported features is presented (Table 4) in comparison with other BS aligners.

**Table 4:** Features supported by BS-Seeker2, Bismark and BSMAP

	**BS-Seeker2**	**Bismark**	**BSMAP**
Support local alignment	Yes	No	No
Tailored for one restriction enzyme RRBS	Yes	No	Yes
Map to reduced representation genome for RRBS	Yes	No	No
Option for removing un-converted reads	Yes	No	No
Tailored for double-restriction enzyme RRBS	Yes	No	No
# of supported input formats	4	2	3
# of supported output formats	3	1	3
Build-in adapter removing function	Yes	No	Yes
Generate wiggle file for methylation levels	Yes	No	No
Report reads coverage for AT	Yes	No	No
Able to manipulate all the parameters of Bowtie(2)	Yes	No	-
Programming language	Python	Perl	C++
Mapping strategy	3-letter	3-letter	wild-card
Available in Galaxy Toolshed	Yes	Yes	Yes
Gapped alignment	Yes	Yes	Yes
Call methylation for CG	Yes	Yes	Yes
Support directional/non-directional sequencing	Yes/Yes	Yes/Yes	Yes/Yes
Support Single-end/Paired-end sequencing	Yes/Yes	Yes/Yes	Yes/Yes

## Discussion

With local alignment, BS-Seeker2 is more capable of mapping “damaged” reads from a bad library with continuous sequencing errors or 3’ end contaminations. Although both BS-Seeker2 and BSMAP consider restriction site information for mapping RRBS-generated reads, BS-Seeker2 is able to process any combination of enzymes, such as double-enzyme digestion protocols (4), facilitating the experiment design on various enzymes. In the real data, a small portion of RRBS-generated reads could originate from regions outside the RR genome, so that they could only be mapped when using the whole genome index. However, these reads are usually not of interest for RRBS data and are best left out.

RRBS libraries tend to have short reads contaminated with adapters in the sequenced reads, and the current *in silico* adapter trimming approach is not always effective to remove them. As a result of evaluation, BS-Seeker2 with local alignments is able to map the most reads than other tools in the comparison.

Both Bowtie and the ‘end-to-end’ mode of Bowtie2 require reads to be fully mapped on the genome. Bowtie utilizes a single-seed method and evaluates the number of mismatches, while Bowtie2 utilizes a multiple-seed method and reports the best hit according to the score calculated by dynamic programming. Generally Bowtie is faster than Bowtie2 but less sensitive for long reads. The ‘local alignment’ mode of Bowtie2 allows reads to be partially mapped by trimming 5’ or 3’ ends during mapping, and BS-Seeker2 in ‘local alignment’ mode will report the hit with the unique best scores, which should exceed the minimum score (defined by the parameter “--score-min” of Bowtie2). The ‘local alignment’ mode requires more computing time due to the dynamic programming, and is effective for aligning reads sequenced with adapters or continuous errors. The ‘local alignment’ mode assumed the true originations of the reads are included in the reference genome, thus it could be possible to introduce false positive if reads are not from the reference genome.

Lastly, we provide some suggestions for the optimal use of BS-Seeker2. For short reads, which usually are of high quality, choosing BS-Seeker2 coupled with Bowtie is enough to achieve a high mappability and is also time efficient. For gapped alignments, BS-Seeker2 with Bowtie2 is the best choice. For long reads with lower quality at the 3’ end, or data where some tiles have low sequence quality in several cycles, Bowtie2 in local alignment mode will achieve higher mappability but requires slightly longer CPU times. The ‘end-to-end’ mode of Bowtie2 could work best for some specific libraries. For example, the ‘multiple seed’ strategy could map more reads in a data set with low sequencing quality at the 5’ ends as it will have more chances to generate a unique hit by searching more seeds.

## Conclusions

We provide a BS alignment pipeline, BS-Seeker2, for fast and accurate mapping of BS reads from various types of library. We improved BS-Seeker2 by utilizing multiple short-read mapping aligners, supporting gapped mapping and local alignment and building special indexes for handling RRBS data. Our comparisons with respect to two other popular BS aligners showed that BS-Seeker2 has a comparable performance on WGBS data and outperforms on RRBS data with the others.

## Availability and requirements

Project name: BS Seeker 2.

Project home page: http://pellegrini.mcdb.ucla.edu/BS_Seeker2/.

Operating system(s): Linux/Mac OS.

Programming language: Python 2.6 + .

Other requirements: pysam package, Bowtie/Bowtie2.

License: MIT License.

Any restrictions to use by non-academics: No.

## Abbreviations

WGBS: Whole genome bisulfite sequencing; RRBS: Reduced represented bisulfite sequencing; BS aligner: Bisulfite sequencing aligner.

## Competing interests

The authors declare that they have no competing interests.

## Authors’ contributions

WG, PF, SC, XS, MQZ, PYC and MP are responsible for design of BS-Seeker2. WG, PYC and MP are responsible for the conception and writing of the manuscript. WG, PF and PYC are responsible for the implementations of BS-Seeker2. WG, WY, PF and PYC are responsible for the configurations of tools on Galaxy toolshed. WY is responsible of maintaining the UCLA Galaxy server of BS-Seeker2. All authors read and approved the final manuscript.

## Supplementary Material

Additional file 1 Pdf file contains Supplementary Methods, Figure S1-S5, Table S1 and descriptions of Supplementary Datasets. Click here for file
