# Supplementary material for: BS-Seeker2: a versatile aligning pipeline for bisulfite sequencing data
Source: BMC Genomics. 2013 Nov 10;14:774. doi: 10.1186/1471-2164-14-774 (PMC3840619; doi:10.1186/1471-2164-14-774)
Supplement: Additional file 1 — Pdf file contains Supplementary Methods, Figure S1-S5, Table S1 and descriptions of Supplementary Datasets. [file 1471-2164-14-774-S1.pdf]

BS-Seeker2: a versatile aligning pipeline for bisulfite sequencing data

Weilong Guo, Petko Fiziev, Weihong Yan, Shawn Cokus, Xueguang Sun, Michael Q Zhang, Pao-Yang Chen, Matteo Pellegrini

Supplemantary Methods

The sequencing error model based on real data

Reads were first mapped to indexes by BS-Seeker2-bowtie with 5 mismatches at most. The sequences of the mapped reads were compared with the genome sequences. The sequencing error rate by cycle was calculated as (# of mapped reads with mismatches) / (# of mapped reads). Sequencing error models were generated for both WGBS and RRBS.

In the simulation data with sequencing error, the sequencing errors were added to each cycle according to the generated model. The sequencing error rate on each cycle in the simulated model was supposed to be independent, thus continuous sequencing errors or indels would not be simulated.

Region selection of fragment lengths for RR genome

For mapping RRBS real data, selecting an appropriate region of fragment sizes would be important to both mappability and accuracy. We mapped the RRBS reads to the whole genome using BS-Seeker2-Bowtie and studied the length distribution of the fragments where the exactly matched reads located, and draw the distribution of fragment lengths (Figure S5). Then the region [20bp, 400bp] was selected for defining a RR genome. We found that about 2.5% reads are mapped to regions outside the RR genome.

Commands for testing aligners

For WGBS : single-end:

```
# WGBS | BS-Seeker2 | bowtie 2 | local alignment
python bs_seeker2-align.py -i WGBS.fa -m 5 --aligner=bowtie2 -f bam -g mm9.fa -t N --bt2-p 1
# WGBS | BS-Seeker2 | bowtie 2 | end-to-end
python bs_seeker2-align.py -i WGBS.fa -m 5 --aligner=bowtie2 -f bam -g mm9.fa -t N --bt2--end-to-end --bt2-p 1
# WGBS | BS-Seeker2 | bowtie
python bs_seeker2-align.py -i WGBS.fa -m 5 --aligner=bowtie -f bam -g mm9.fa -t N --bt-p 1
# WGBS | Bismark | bowtie 2
bismark -f mm9_bt2_bismark --bowtie2 -L 15 -D 50 --score_min L,-0.6,-0.6 WGBS.fa --temp_dir=/tmp
# WGBS | Bismark | bowtie 1
bismark -f mm9_bt1_bismark WGBS.fa -e 200 --temp_dir=/tmp
# WGBS | BSMAP
```

For RRBS:

```
# RRBS | BS-Seeker2 | bowtie 2 | local
python bs_seeker2-align.py -i RRBS.fa -m 5 --aligner=bowtie2 -f bam –g mm9.fa -t N -r --low=20 --up=400 -a adapter.txt --am=2 --bt2-p 1
# RRBS | BS-Seeker2 | bowtie 2 | end-to-end
python bs_seeker2-align.py -i RRBS.fa -m 5 --aligner=bowtie2 -f bam -g mm9.fa -t N -r --low=20 --up=400 -a adapter.txt --am=2 --bt2--end-to-end --bt2-p 1
# RRBS | BS-Seeker2 | bowtie
python bs_seeker2-align.py -i RRBS.fa -m 5 --aligner=bowtie -f bam -g mouse_mm9.fa -t N -r --low=20 --up=400 -a adapter.txt --am=2 --bt-p 1
# RRBS | Bismark | bowtie 2
trim_galore -rrbs -a AGATCGGAAGAGCACACGTCTGAACTCCAGTCA RRBS.fq;
bismark -q mm9_bt2_bismark --bowtie2 -L 15 -D 50 --score_min L,-0.6,-0.6 RRBS_trimmed.fq --temp_dir=/tmp
# RRBS | Bismark | bowtie
trim_galore -rrbs -a AGATCGGAAGAGCACACGTCTGAACTCCAGTCA RRBS.fq;
bismark -q mm9_bt1_bismark RRBS_trimmed.fq -e 200 --temp_dir=/tmp
# RRBS | BSMAP
bsmap -a RRBS.fa -d mm9.fa -v 0.05 -w 2 -D C-CGG -A AGATCGGAAGAGCACACGTCTGAACTCCAGTCA -r 0 -p 2
```

For WGBS, Paired-end data:

```
# Pair-end | BS-Seeker2 | bowtie 2 | local
python bs_seeker2-align.py -1 end1.fa -2 end2.fa -m 2 --aligner=bowtie2 -f bam –g hg18.fa -
t N --bt2-p 1 --bt2-I 0 --bt2-X 600
# Pair-end | BS-Seeker2 | bowtie 2 | end-to-end
python bs_seeker2-align.py -1 end1.fa -2 end2.fa -m 3 --aligner=bowtie2 -f bam -g hg18.fa -
t N --bt2--end-to-end --bt2-p 1 --bt2-I 0 --bt2-X 600
# Pair-end | BS-Seeker2 | bowtie
python bs_seeker2-align.py -1 end1.fa -2 end2.fa -m 3 --aligner=bowtie -f bam -g hg18.fa -t
N --bt-p 1 --bt-I 0 --bt-X 600
# Pair-end | Bismark | bowtie 2
bismark -q hg18_bt2_bismark -1 end1.fa -2 end2.fa --bowtie2 -L 15 -D 50 --score_min L,-
0.6,-0.6 --temp_dir=/tmp -X 600 -I 0
# Pair-end | Bismark | bowtie
bismark -q hg18_bt1_bismark -1 end1.fa -2 end2.fa -f -e 120 -X 600 -I 0 --temp_dir=/tmp
# Pair-end | BSMAP
bsmap -a end1.fa –b end2.fa -d hg18.fa -v 3 -m 0 -x 600 -r 0 -w 2 -p 2
```

Note: The above commands are used for comparing the three aligners by specifying 2 threads in practice. BS-Seeker2 will create two bowtie instances for aligning (two strands), and the parameter "--bt-p 1" and "--bt2-p 1" will ensure that each bowtie instance runs with 1 thread. Thus BS-Seeker2 will align reads with 2 threads in total. Bismark will also create two bowtie instances for aligning, and the default parameter "-p 1" will ensure each instance run with 1 thread. Thus Bismark will align reads with 2 threads in total. The parameter "-p 2" of BSMAP will ensure BSMAP runs with 2 threads in total.

Formats of CGmap and ATCGmap files

CGmap file

Format description for each column:

(1) chromosome
(2) nucleotide on Watson (+) strand
(3) position
(4) context (CG/CHG/CHH)
(5) dinucleotide-context (CA/CC/CG/CT)
(6) methyltion-level = #-of-C / (#-of-C + #-of-T)
(7) #-of-C (methylated)
(8) (#-of-C + #-of-T) (all cytosines)

Format example:

```
chr1    C       702973  CHH     CC      0.0     0       4
chr1    G       703153  CHH     CC      0.0     0       6
chr1    G       703154  CHH     CC      0.167   1       6
chr1    G       703157  CG      CG      1.0     6       6
chr1    G       703160  CHG     CA      0.0     0       6
chr1    G       703169  CG      CG      0.833   5       6
chr1    G       703173  CHG     CA      0.0     0       6
chr1    G       703181  CG      CG      1.0     6       6
```

ATCGmap file

Format description for each column:

(1) chromosome
(2) nucleotide on Watson (+) strand
(3) position
(4) context (CG/CHG/CHH)
(5) dinucleotide-context (CA/CC/CG/CT)
(6) - (10) plus strand
(6) # of reads from Watson strand mapped here, support A on Watson strand
(7) # of reads from Watson strand mapped here, support T on Watson strand
(8) # of reads from Watson strand mapped here, support C on Watson strand
(9) # of reads from Watson strand mapped here, support G on Watson strand
(10) # of reads from Watson strand mapped here, support N
(11) - (15) minus strand
(11) # of reads from Crick strand mapped here, support A on Watson strand and T on Crick strand
(12) # of reads from Crick strand mapped here, support T on Watson strand and A on Crick strand
(13) # of reads from Crick strand mapped here, support C on Watson strand and G on Crick strand
(14) # of reads from Crick strand mapped here, support G on Watson strand and C on Crick strand
(15) # of reads from Crick strand mapped here, support N
(16) methylation_level = #C/(#C+#T) = C8/(C7+C8) for watson strand, =C14/(C11+C14); "nan" means none read support C/T at this position.

Format example:

```
chr1  T   227045  --    --    0     22    0     0     0     0     0     0     0  na
chr1  C   227046  CHH   CA    0     22    0     0     0     0     0     0     0  0.0
chr1  A   227047  --    --    22    0     0     0     0     0     0     0     0  na
chr1  C   227048  CHH   CC    3     19    0     0     0     0     0     0     0  0.0
chr1  C   227049  CHH   CC    0     22    0     0     0     0     0     0     0  0.0
chr1  C   227050  CHH   CC    0     22    0     0     0     0     0     0     0  0.0
chr1  C   227051  CHG   CT    0     21    1     0     0     0     0     0     0  0.045
chr1  T   227052  --    --    0     22    0     0     0     0     0     0     0  na
chr1  G   227055  CHH   CC    0     0     0     22    0     0     0     0     0  na
```

SUPPLEMENTARY DATA STATEMENTS

We provide data sets used in this study on our websites (http://pellegrini.mcdb.ucla.edu/BS_Seeker2/).

Data sets for testing aligners

Simulation data set without sequencing error: Dataset 1 (WGBS, single-end, fasta), Dataset 2 (WGBS, paired-end, fasta) and Dataset 3 (RRBS, fasta).

Simulation data set with sequencing error: Dataset 4 (WGBS, single-end, fasta), Dataset 5 (WGBS, paired-end, fasta) and Dataset 6 (RRBS, fasta).

Real sequencing data: Dataset 7 (WGBS, single-end, fasta), Dataset 8 (WGBS, paired-end, fasta) and Dataset 9 (RRBS, fasta).

Data sets for phage analysis

Data set used for validation on phage DNA: Dataset 10 (qseq format).

Data sets to validate filtering unconverted read function

Data sets used to validate filtering unconverted read function: Dataset 11 (Sample A, qseq format) and Dataset 12 (qseq format).

Supplemantary Figures

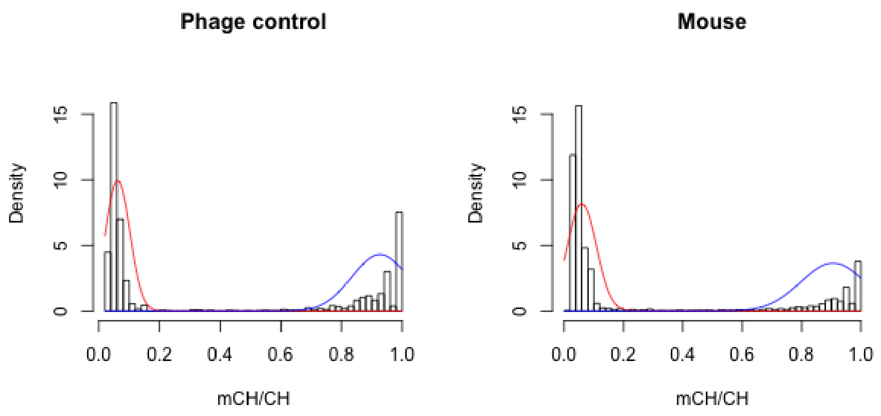

Figure S1. Distribution of the unconverted ratio of CH sites (H = A, C, T) in phage DNA reads (left) and mouse liver DNA reads (right), and each read has at least one CH site unconverted. Phage DNA is free of DNA methylation and used as a control. Mouse liver DNA is supposed to be very low in non-CpG methylation. The distribution chart indicates two different categorises: sporadic (red) and dense (blue) groups. BS-Seeker2 provides an option for removing reads with densely un-converted non-CpGs.

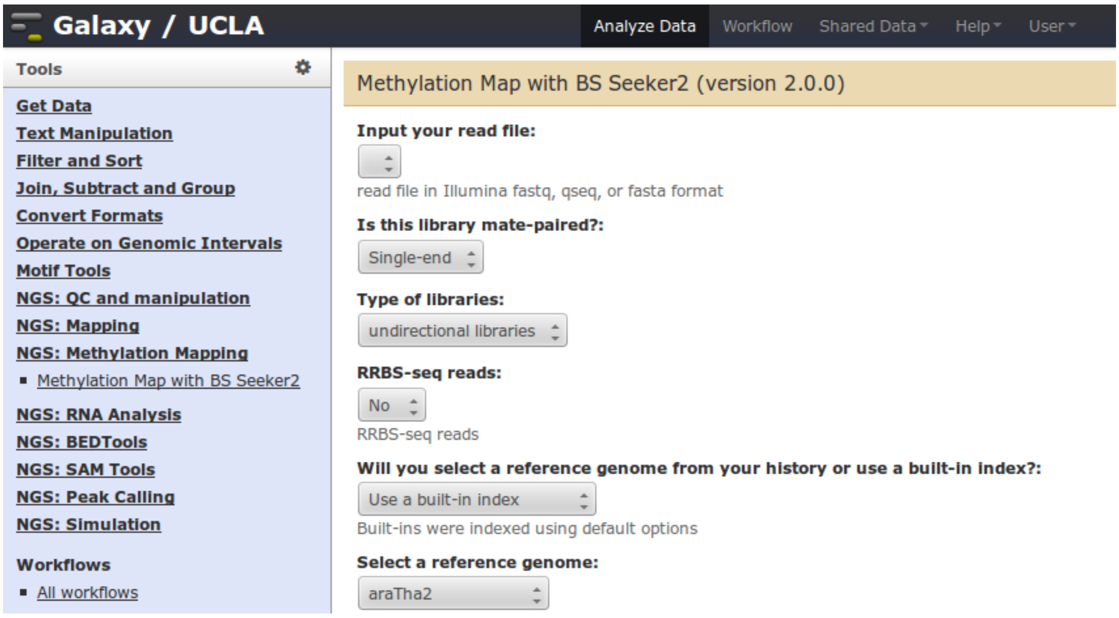

Figure S2. Implementation of BS-Seeker2 in Galaxy (UCLA version)

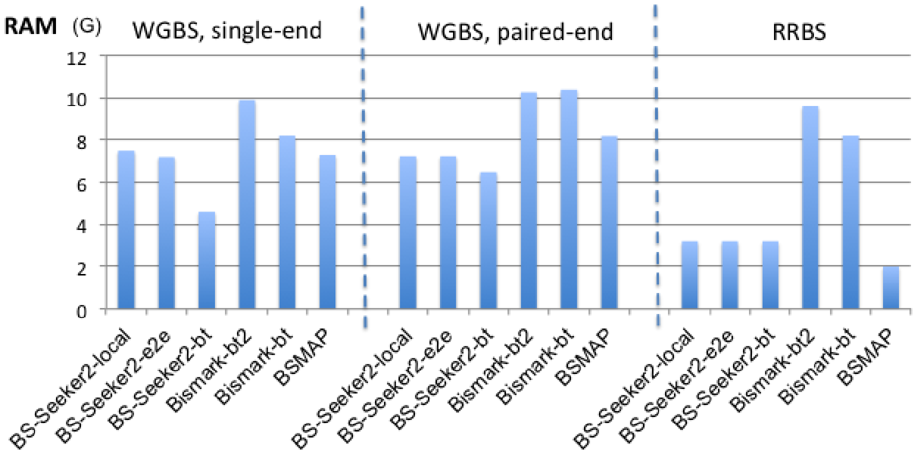

Figure S3. The RAM costs of the aligners for mapping 100k real sequenced reads from WGBS/single-end data set (left), WGBS/paired-end data set (middle), and RRBS data set (right).

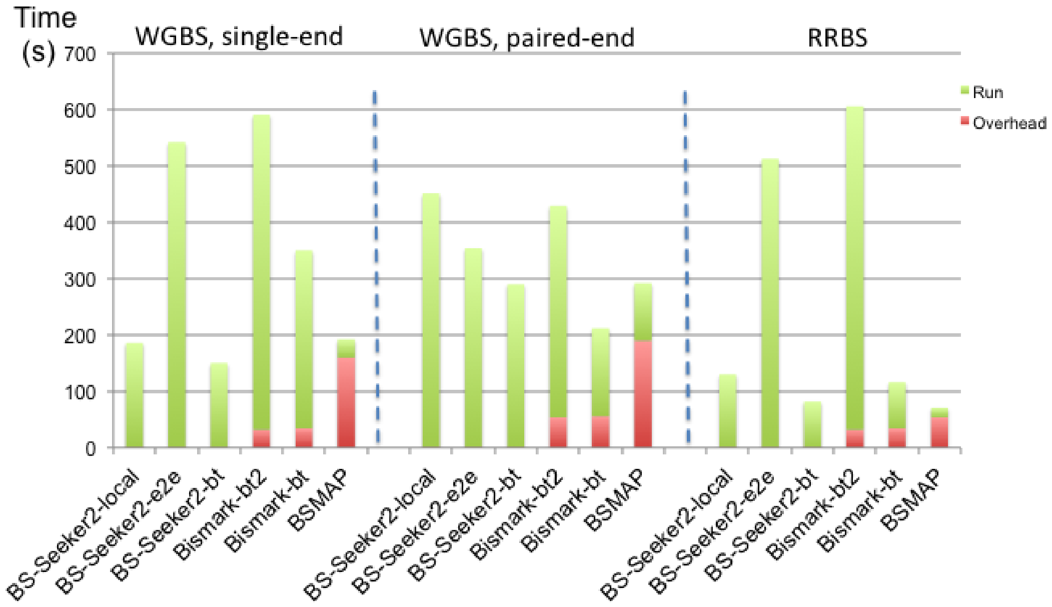

Figure S4. The CPU time costs of the aligners for mapping real reads from WGBS/single-end data set (left), WGBS/paired-end data set (middle), and RRBS data set (right). The height of whole bar shows the time cost for mapping 100k reads. The height of red bar shows the time cost for mapping only one read, which could be regarded as the overhead of the programs.

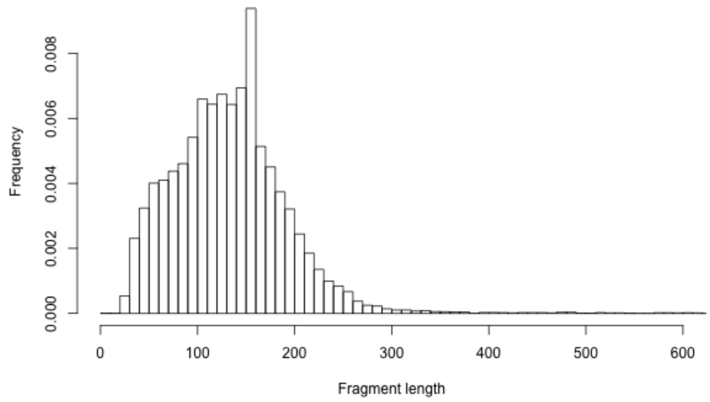

Figure S5. Distribution of the fragment lengths from the real RRBS library. Fragment lengths are estimated from the mapped results on whole genome using bowtie.

Supplemantary Tables

Table S1 - Performance comparison of BS aligners on paired-end bisulfite sequencing data

map=mappability, acc=accuracy, local=local alignment mode, e2e=end-to-end alignment mode. The real pair-end data is from SRR306438. The 100k reads are trimmed to 60bp on each mate and used for comparison. The sequencing error model for simulation data is generated according to the real data. Al the datasets were mapped to the human reference genome (hg18).

	Sequencing error		BS-Seeker2			Bismark		BSMAP
			bowtie2		bowtie	bowtie2 (e2e)	bowtie	
			local	e2e				
simulation	no	map	95.62%	95.29%	95.06%	93.07%	94.90%	90.03%
		acc	99.30%	99.41%	99.56%	99.97%	99.09%	99.03%
	yes	map	94.08%	94.79%	94.74%	93.14%	93.31%	89.29%
		acc	99.17%	99.30%	99.51%	99.94%	99.15%	98.94%
Real		map	68.38%	43.21%	42.41%	48.11%	42.40%	42.88%
